# Supplementary material for: Does entropy modulate the prediction of German long-distance verb particles?
Source: PLoS One. 2022 Aug 4;17(8):e0267813. doi: 10.1371/journal.pone.0267813 (PMC9352069; doi:10.1371/journal.pone.0267813)
Supplement: S1 Appendix — (PDF) [file pone.0267813.s001.pdf]

Appendix S1 Detail on pre-registrations.

Experiment 1 was pre-registered on OSF `https://osf.io/qbna2`. Deviations from this pre-registration were: i) the number of critical trials analysed was 40 and not 44 as pre-registered (4 items were excluded for not meeting the criteria described under *Materials*); ii) we analysed a region of electrodes rather than the pre-registered single electrode, as region analyses are standard in the literature (see Appendix S2 for a comparison of the single-electrode vs. region analysis results, in which our conclusions would have been the same); iii) we used regularising priors with a standard deviation of $1\mu V$ ($Normal(0,1)$) which assumed that ERP effects would vary with 95% probability between $-2\mu V$ of zero. In contrast, the pre-registered priors had standard deviations of 5 and even $10\mu V$, which are less plausible effect sizes based on previous psycholinguistic ERP research. Moreover, regularising priors better constrain the statistical model [1–4]; iv) we added hypotheses and analyses concerning the PNP whose existence we were unaware of at the time of the pre-registration; v) we used Bayes factors to quantify evidence for/against effects rather than the pre-registered proportion of the posterior distribution that was above zero, since the latter does not rule out a null effect or an effect in the opposite direction if zero is a plausible value in the posterior [5].

Experiment 2 was pre-registered at `https://osf.io/y6k2d`. The deviations to the pre-registration were: i) the number of critical trials was 50 rather than 54; ii) we used regularising priors rather than the same priors pre-registered in Experiment 1; iii) we added Bayes factor analyses to inform inference.

References

1. Gelman A. Prior Choice Recommendations; 2020. Available from: `https://github.com/stan-dev/stan`.

2. Chung Y, Gelman A, Rabe-Hesketh S, Liu J, Dorie V. Weakly Informative Prior for Point Estimation of Covariance Matrices in Hierarchical Models. Journal of Educational and Behavioral Statistics. 2015;40(2):136–157. doi:https://doi.org/10.3102/1076998615570945.

3. Gelman A, Jakulin A, Pittau MG, Su YS. A weakly informative default prior distribution for logistic and other regression models. Annals of Applied Statistics. 2008;2(4):1360–1383. doi:10.1214/08-AOAS191.

4. Gelman A, Simpson D, Betancourt M. The Prior Can Often Only Be Understood in the Context of the Likelihood. Entropy. 2017;19(10):555. doi:10.3390/e19100555.

5. Wagenmakers EJ, Lee MD, Rouder JN, Morey RD. The Principle of Predictive Irrelevance, or Why Intervals Should Not be Used for Model Comparison Featuring a Point Null Hypothesis. Annals of Theoretical Psychology book series. 2020;16:20.
