# Supplementary material for: Does entropy modulate the prediction of German long-distance verb particles?
Source: PLoS One. 2022 Aug 4;17(8):e0267813. doi: 10.1371/journal.pone.0267813 (PMC9352069; doi:10.1371/journal.pone.0267813)
Supplement: S2 Appendix — (PDF) [file pone.0267813.s002.pdf]

Appendix S2 Pre-registered single electrode analyses for Experiment 1.

The dependent variables for Experiment 1 deviated from the pre-registration where we proposed analysing a single electrode as a way of reducing averaging, which can artificially reduce variance [1]. However, the disadvantage of analysing single electrodes is that ERP components are not so highly focal as to appear at one electrode, and so the already high signal-to-noise ratio inherent in ERP can drown out genuine effects or create the illusion of an effect where there is none [2, 3]. For this reason, later in Experiment 2, we pre-registered and analysed a region of electrodes based on the results of Experiment 1. To make the presentation of results between both experiments consistent, we presented region analyses for both experiments. However, for transparency, we present here a comparison of the posterior estimates obtained via the pre-registered, single electrode analysis of Experiment 1 with those obtained via the region analysis; see Figure S2.

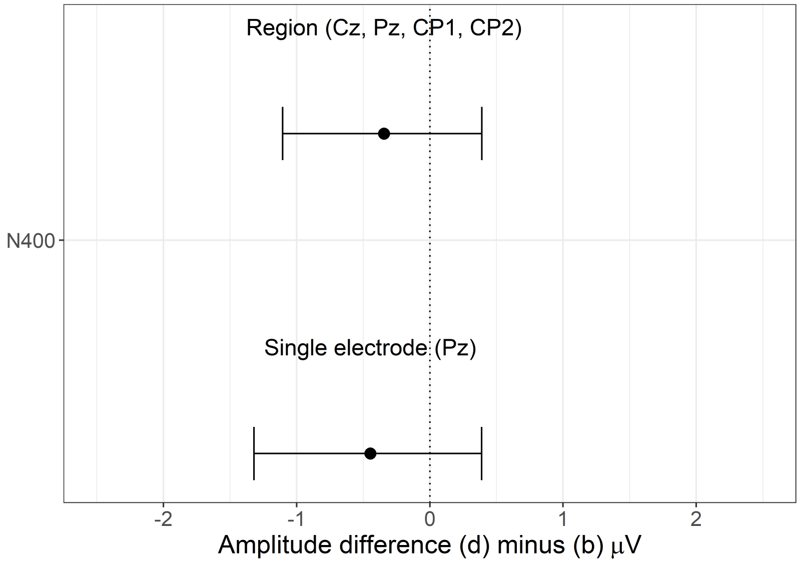

Fig 1. Comparison of the results of the pre-registered single-electrode analysis of the N400 in Experiment 1 with the region-based analysis presented in the main manuscript. Points and errorbars represent the means and 95% credible intervals estimated for the comparison of 1-particle and 2+particle violations in the N400 time window. Both analysis approaches yield comparable estimates, confirming that the region analysis used in the main manuscript did not lead to more (or less) favourable conclusions about the N400.

References

1. Barr DJ, Levy R, Scheepers C, Tily HJ. Random effects structure for confirmatory hypothesis testing: Keep it maximal. Journal of Memory and Language. 2013;68(3):255–278. doi:10.1016/j.jml.2012.11.001.

2. Luck SJ. Ten Simple Rules for Designing and Interpreting ERP Experiments. In: Handy TC, editor. Event-related Potentials: A Methods Handbook. MIT press; 2005. p. 17–32.

3. Luck SJ, Gaspelin N. How to Get Statistically Significant Effects in Any ERP Experiment (and Why You Shouldn't). Psychophysiology. 2016;4424. doi:10.1111/psyp.12639.
