# Supplementary material for: Does entropy modulate the prediction of German long-distance verb particles?
Source: PLoS One. 2022 Aug 4;17(8):e0267813. doi: 10.1371/journal.pone.0267813 (PMC9352069; doi:10.1371/journal.pone.0267813)
Supplement: Attachment — Submitted filename: Response_to_reviewers.pdf [file pone.0267813.s003.pdf]

Itemised responses to reviewers

Editor comments:

1. I would invite the authors to carefully consider all the suggestions advanced by both Reviewers. I concur that the paper in its present form is well-written and worth to be published. However, I am in line with Reviewer #1 that "this paper uses statistics from the future, but ERP analysis/visualisation from the past". Following the Reviewers' points the paper can be largely improved.

   Thank you and with respect to ERP visualisation and analysis, please see our responses to Reviewer 1.

Reviewer 1:

Stone et al. present an ERP study on an interesting topic, the role of uncertainty in lexical prediction (i.e. commitment to a specific word). They approach it by cleverly exploiting long-distance verb particles in German. Results of the initial experiment turned out inconclusive, so the authors (laudably) ran a replication with a large sample size. In the end, the results were still inconclusive (statistically speaking at least, in practice _I think_ we can at least conclude there was not a lot of evidence for the hypothesis). However, the authors fully embraced this uncertainty using sophisticated and comprehensive Bayesian analyses. They also make some interesting suggestions as to why the results turned out the way they did.

Overall, I think this is a good study: smart design, rigorous methods, appropriate (in)conclusions. I have no major comments. To my mind, it could in principle be published as-is. However, I do have _some_ questions and comments that the authors may want to engage with. Hopefully (some of) it will be useful in finalizing the manuscript, in case of some very minor revisions.

2. <u>Zero-probability words</u>
   A key assumption in the design is that the low and high uncertainty condition, implausible particles are “zero probability” – defined in a cloze task – and hence equally unlikely. As such, any potential difference can be attributed to the uncertainty, not predictability.

   However, we know that predictability effects are logarithmic: comprehenders are sensitive to subtle differences between seemingly small probabilities (e.g. 0.005 vs 0.0005). Words with such low probabilities will be practically never produced in a cloze norming task, and hence are seemingly equally unlikely, although they are not in fact of equal predictability.
   One instance where this may apply is in the amenability to repair. The authors write that:

   “German particles are identical to prepositions and thus readers may have attempted to revise the particle as a preposition, e.g .”carried his lecture [with determination] on”. The large P600 in both experiments may suggest that such reanalysis was attempted” (735-737)

Could it be that some “implausible” articles are more open to such alternative interpretations than others? If so, this would arguably reflected in their predictability: implausible words that can be “saved” with alternative interpretations will be less unlikely. If the materials were English, this would be easy enough to assess: use a modern language model to quantify the unpredictability of the implausible articles. Perhaps this could capture additional variance, and strengthen the inference. For German, I am not sure if sufficiently competent LMs exist though (if so, they will probably be on the modelhub at hugginface though).

To be clear, these are just suggestions and I don’t think any such additional analyses are required for acceptance. However, I was struck by the fact that the authors so uncritically use the notion of “zero probability”, while also appealing to the concept of probabilistic/graded pre-activation, and error-based accounts of N400 (e.g Fitz & Chang, 2019), where this error is based on a softmax that will almost never return a predictability of absolutely zero?

Thank you for raising this interesting point. There are two issues here: first, what is “probable” before the particle has been seen, and second, what can be done once the implausible particle is encountered. When we used the term ‘zero probability’, we had in mind what lexical items were probable before the particle was seen. Assuming a link between probability and preactivation, we assumed preactivation of an implausible particle would be as good as zero. However, as the Reviewer suggests, it could indeed be that probability/preactivation is never truly zero; for example, implausible particles may receive some spreading activation simply because they belong to the category “particle”, affecting both the N400 and PNP. It could even be that the particle’s lexical entry receives some activation from expectation of a preposition, although to what degree particles and prepositions share memory representations is an interesting question. If it were possible to activate a preposition via a particle, then once our implausible particle was encountered, it may have been easier to revise some implausible particles as prepositions, affecting the PNP. This would absolutely be interesting to model, but beyond the scope of the current paper. Nonetheless, in light of this discussion, when we first mention “zero probability” in the paper, we have added the following clarification on p10, line 230:

> “We use the term ``zero probability'' when referring to the implausible particles to reflect the fact that the sentence context is highly unlikely to preactivate implausible particles in memory as they would be meaningless in the context. However, there exists evidence from the N400 demonstrating that readers can be sensitive to differences within the very low end of the probability spectrum, including for implausible words (Szewczyk et al., 2021). These activations are unlikely to be captured by a cloze test and thus have a cloze probability of zero, but their preactivation may still affect ERP amplitude. This would be a caveat to our ``matched zero probability” target particles. The contribution of very low probability words to entropy and ERP amplitude needs future investigation, but for the current study we use the term “zero probability” to refer broadly to a situation where, for the purposes of studying the strength of people’s probabilistic representations of the context of a sentence, the contribution of preactivation from an implausible particle is effectively zero.”

3. Measurement and use of entropy
   The authors extensively appeal to the concept of entropy, which (rightly so, IMO) they suggest is a better formalization of (inverse) constraint than highest cloze probability. But if I'm not mistaken, they only use a categorical distinction between "number of plausible particles" in their statistical comparison. Why is this? Why not use the empirical entropy? Surely the entropy from the cloze will be imperfect, but it would contain more information about the distribution than the simple distinction between 1-particle vs 2+-particle?

   I'm sure the authors have good statistical reasons for this, but maybe they can give them?

   It would indeed have been nicer to be able to use entropy at the particle as a measure of constraint. The rationale for not doing this was that we were unsure about the functional relationship between entropy and ERP amplitude, i.e. whether it was linear or something else. We would have had difficulty verifying the form of the relationship because rather than an even spread of entropy values between conditions, we had two clusters: one around 0.3 and one around 0.8. We therefore felt that using a categorical predictor was a better representation of the data, even if the model still assumed a linear relationship. We've added this explanation to p18, line 432:

   > "The predictor `number of plausible particles' was effect contrast coded to reflect our expectation for larger ERPs at 1-particle violations: 0.5 (1-particle), -0.5 (2+particle). The decision to use a categorical predictor rather than raw entropy was made because the precise functional relationship between entropy and ERP amplitude was unknown, and we felt unable to safely assume it was linear."

4. Uncertainty and the notion of lexical prediction
   This is point is more of a conceptual question than a comment.

   The authors investigate the conditions under which comprehenders make a lexical prediction, here defined as an all-or-none commitment to a specific word. Since such a commitment is risky and can lead to "costly misprediction", the hypothesis was that comprehenders only make such a commitment when the entropy is low.

   Now, this all seems perfectly reasonable, and well in line with how lexical prediction has long been conceptualized in psycholinguistics (e.g. van Petten and Luka, 2012). But I was wondering: is there any evidence that comprehenders actually make such commitments? I'm asking because in the introduction, the authors seem to take for granted that 'lexical prediction' (i.e. commitment) and probabilistic/graded pre-activation both exist in parallel. But is there any evidence for this (ignoring visual world tasks, that enforce commitment)? Isn't the evidence equally consistent with the idea that all predictability effects can be attributed to a kind of graded preactivation (with more or less certainty)?

   As far as we know, there is not yet conclusive evidence that prediction is a commitment rather than just strong activation, or vice versa, so it's an interesting and open question. One goal of our experiment had been to try and show evidence for commitments when entropy is low vs. high,

although without a graded entropy predictor (see point 3 above) it would have been difficult to disentangle whether we had captured a separate commitment process or just the extreme end of a graded preactivation scale. We have added some background to the open question of commitment vs. graded activation on p4, line 69:

> "Commitment to a specific sentence interpretation refers to one account of the predictive mechanism in sentence processing where a specific interpretation might be stored in working memory (Pickering & Gambi, 2018; Kuperberg & Jaeger, 2016; Ness & Meltzer-Asscher, 2018; Lau, 2018). However, the mechanisms underlying predictive processing are by no means resolved. An alternative account is that the level of preactivation alone is sufficient to explain predictability effects (Staub, 2015). This is supported by the success of metrics such as surprisal that measure the probability of a word given its context---which could be assumed to be linked to word preactivation---in accounting for variability in predictability-related empirical effects (Hale, 2006; Levy, 2008; Smith & Levy, 2013; Frank et al., 2015; Lowder et al., 2018; Szewczyk & Federmeier, 2021). On the other hand, it has been shown that suprisal alone is not sufficient to explain all differences in reading times or the N400 (van Schijndl & Linzen, 2021; Michaelov & Bergen, 2020). In the case of reading times, this was taken to suggest the need of an additional explanatory factors such as reanalysis or revision (van Schijndl & Linzen, 2021). Assuming that reanalysis or revision can only be triggered if a commitment has been made, such findings could be taken as evidence that predictions can involve commitments."

5. And relatedly, if graded/probabilistic prediction is all there is, would the authors still expect an effect of entropy, over and above surprisal? Why/why not?

Even if graded activation is all there is to predicting an upcoming word, the ERP reflects a number of processing stages where the predictive process may have an impact. Depending on the processing stage, surprisal and entropy may become better or worse predictors of amplitude; for example, we would perhaps not expect to see an entropy effect when predictive processing is driven by lexical activation levels, such as in the N400 window. Surprisal (or cloze probability) would therefore be a better predictor here, and this is what we observe empirically (Federmeier et al., 2007; Frank et al., 2015; Kuperberg et al., 2020; Kutas & Hillyard, 1984). But when predictive processing influences the degree or type of revision that might be needed to a broader probabilistic representation, such as in the post-N400 window, then perhaps entropy will be a better predictor, as it may reflect (for example) the range of preactivated alternative parses. In a forthcoming registered report, we actually show that the post-N400 window of the ERP is affected by entropy and not cloze probability, and vice versa in the N400 window (Stone et al., 2020). This echoes previous studies of constraint vs. cloze probability (Federmeier et al., 2007; Kuperberg et al., 2020) (cloze probability in these studies could be taken to approximate surprisal).

Even-more-minor comments:

6. EEG channel selection.
   The authors use an ROI to define the N400. This apparently diverges from their preregistration but it seems a good choice to me. The selected channels also seem reasonable. However, what is the motivation for using these particular channels? Is it a prior study, if so maybe they can give it?

   For Experiment 1, our ROIs were chosen post-hoc region based on the pattern of amplitudes, but these ROIs were generally consistent with previous research on the N400 and PNP. The PNP is sometimes seen more frontally, but given the different syntactic/lexical construction (particle verbs) and lab-based differences in data acquisition, we felt we were justified in using the post-hoc ROI choice from Experiment 1. We have added a sentence on this point to the manuscript on p18, line 425:

   > "For Experiment 1, these regions were chosen post hoc after we deviated from the pre-registered single-electrode analysis (see Appendix 1 and Appendix 2). The regions were generally consistent with the distribution of the N400 and PNP observed in the predictive processing literature (e.g. Kuperberg et al., 2020, De Long et al., 2005; Federmeier et al., 2007), although some studies observe a more anterior PNP (Kuperberg et al., 2020; Brothers et al., 2020). Since they were chosen post hoc, the regions of interest in Experiment 1 were used as pre-registered regions in Experiment 2."

7. EEG visualization and analysis.
   While I was very impressed with the statistics, I was a bit disappointed by the presentation of the EEG data and results. EEG results only convey point estimates of the mean, no errorbars or other visualisations of statistical consistency. For instance, looking at the red line and blue dashed line in Figure 1a, the plot is equally consistent with a very strong effect in the expected direction, as with no effect. I understand that the authors' statistical analysis is too sophisticated to be accurately conveyed by any single error bar. However, as currently presented the EEG visualisations are really not that informative.

   Again, this is not something that _must_ be fixed before acceptance. However, I did want to say that as a reader, I get the impression that this paper uses statistics from the future, but ERP analysis/visualisation from the past… Maybe something to consider in future projects?

   Confidence intervals have now been added to Figures 1 and 3:

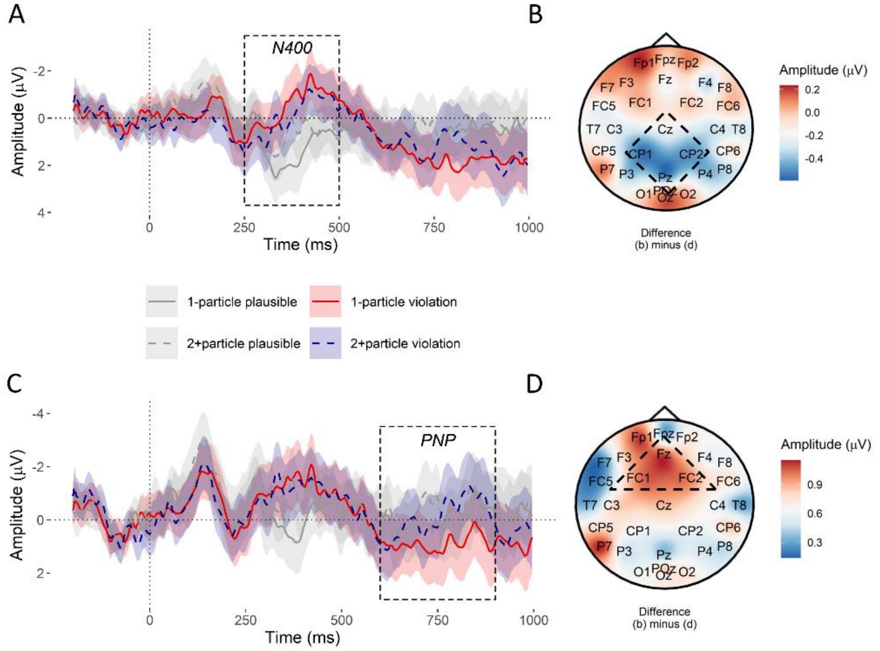

Figure 1.

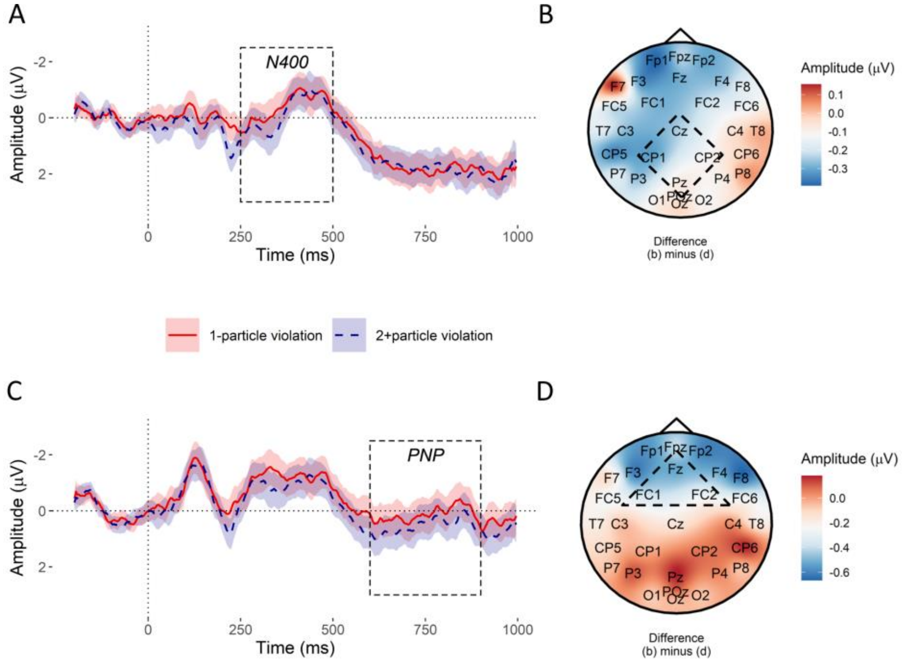

Figure 3.

Reviewer 2:

This paper presents a study with two experiments (the second being a replication of the first) examining the effect of semantic predictability in sentence processing: Do speakers of German predict specific particles in particle-verb constructions, where quite a lot of intervening material can come between the two elements? They compared sentences where either a single particle was semantically plausible versus sentences where more than one particle was plausible. The critical contrast was in cases where a violation occurred, which would ultimately both elicit the ERP components of interest (N400 and PNP). The main question was whether the magnitude of these effects would differ in each case (and in the case of the PNP, whether it would occur at all). On one hand, both may elicit an equal effect—a potentially meaningful null effect, indicating that components such as the N400 are not sensitive to entropy. On the other hand, there may be a difference, indicating both that the N400 is sensitive to entropy, and that such distributional facts are being used by language comprehenders.

First, some positive notes. To my knowledge, the authors are making excellent use of statistical tools to analyze the results of their studies, and the questions that motivate this study are of general interest. At the end of the day, however, the results of this study do not bring us any closer to finding an answer to the very good questions that the authors pose. As demonstrated by the Bayes Factor analysis, the findings are inconclusive. No difference was observed between the critical conditions in either the N400 or PNP, and we cannot have confidence that this is a true null effect. The authors do an excellent job of carefully presenting and discussing this inconclusiveness. I think this is a model for how a paper should handle such results.

What gives me particular pause is that the lack of a conclusive result was highly predictable. The authors report that a power analysis was run prior to Experiment 2 based on the results of Experiment 1, and yet, they still ran a highly underpowered study. Only 1/3 of the number of participants to achieve 70% power were run (Side note: what is the power for the study that was actually run? That is, the power for a 100 person study? This is not reported, but should be). The authors make statements (e.g. in the abstract) that the results were “surprisingly unclear”. I actually do not think, per the authors own power analysis, that this unclarity is at all surprising. Quite the opposite, it was expected. I think this should be more directly addressed.

We appreciate the Reviewer's concern and note that our original discussion of sample size determination for Experiment 2 lacked detail. Despite the predicted lack of power, our goal in conducting Experiment 2 was to provide higher-precision estimates of the effects observed in Experiment 1 and to see to what extent doubling the size of the experiment would improve our ability to quantify evidence for our hypotheses. We felt this contribution was important given the weaknesses in the design of Experiment 1, which may have yielded inaccurate estimates (e.g. note that the sign of the PNP estimate reversed between experiments). Even though we were ultimately unable to find strong evidence for our hypotheses, Experiment 2 thus improves our knowledge over Experiment 1, especially with respect to how one might approach future experiments. We have clarified each of these issues in our discussion of sample size determination on page 24, line 591:

> "To estimate a suitable sample size for Experiment 2, we conducted a power analysis using the data from Experiment 1. Although Bayesian analysis was used for the main analysis, we conducted a frequentist power analysis as it was computationally lighter and serves as a quick ballpark estimate of the sample size needed for obtaining high-precision estimates (Vasishth et al., 2022). The power analysis suggested that even with 300 participants we would only achieve around 70% power. Due to resource constraints, it was only feasible to collect data from around 100 participants. We therefore set a goal of 100 participants as the maximum feasible sample size and redid the power analysis, which estimated around 50% power at this sample size. While likely to be underpowered, our goal with Experiment 2 was to provide higher-precision estimates of the effects observed in Experiment 1 using an improved design, and to see to what extent doubling the size of the experiment would improve our ability to quantify evidence for our hypotheses. This information is useful to future evidence synthesis and in determining how one might approach future experiments."

In the General Discussion, we also discuss a prospective power analysis using the estimates of Experiment 2 to inform the design of a hypothetical future experiment on p31, line 770:

> "With respect to sample size, we again used a frequentist power analysis to gain a rough estimate of the sample size that would be needed for a hypothetical, future experiment using the same experimental design and assuming that the effect was the same size observed in Experiment 2 (Gelman & Carlin, 2014). This prospective power analysis suggested that thousands of participants would be needed to achieve even 30% power, strongly suggesting that the above questions are likely not answerable with this particular design. Compare this to the power analysis conducted using the lower-precision estimates from Experiment 1, which vastly overestimated that we could achieve around 50\% power with just 100 participants."

We would also like to clarify our use of the word 'surprising': what we find surprising about the inconclusive result is that, based on the literature showing that the PNP should not differ in amplitude between two implausible words, the results of Experiment 2 should really have been very clearly in favour of the null hypothesis at our a priori hypothesised effect size (note that the sample size was sufficient to find strong evidence for the standard N400 predictability effect, as well as against very large entropy effect sizes). Given this clear prediction from the literature, it is therefore surprising that with 100 participants, the Bayes factor was not even weakly in favour of the null hypothesis. This suggests something about the difference in study design relative to previous studies, and we propose on p32 beginning line 788 that this may have something to do with having used violating verb particles rather than nouns. In light of the reviewer's suggestion, we have rephrased our statements about "surprising given the sample size" to "surprising given the clear predictions made by the literature" in the abstract and the conclusions.

8. Generally I think this paper would benefit by being re-written in a way that does not lead to any expectation that the main research question will be answered. Perhaps more of a methods paper with a deeper literature review on the components of interest. There are some good hints of what this might look like in the discussion and conclusion. For example, focusing the paper around the interpretation of ERP components rather than around whether language comprehenders use entropy

to make predictions. This would be a more appropriate framing for a paper that lacks interpretable results and cannot actually answer those questions.

We appreciate this suggestion and have added the following sentence to the first paragraph of the Introduction on p3, line 19:

> "Although we did not find a conclusive answer to this question, we report our attempt as a resource for future researchers who may wish to revisit the issue. Furthermore, by reporting the inconclusive results, we avoid contributing to publication bias via the ``file drawer problem" (Rosenthal, 1979)."

The abstract also states that the results were inconclusive. We hope the Reviewer finds this a satisfactory way to frame the paper as an attempt to answer a reasonable question that could have worked, but didn't.

9. Study 2 says 115 were tested, 16 were excluded (4 for not meeting inclusion criteria, 12 for technical issues), but then 100 were included in the analysis. There must be a mistake somewhere, because 115 minus 16 is 99, not 100.

This has been rectified on p24, line 606 (one subject with technical issues was counted twice in error):

> "In total, 115 participants were recruited, 4 of whom were excluded as they did not meet the inclusion criteria. A further 11 were excluded due to technical problems with the EEG recording. This left a total of 100 participants…"

References

Federmeier, K. D., Wlotko, E. W., Ochoa-Dewald, E. D., & Kutas, M. (2007). Multiple effects of sentential constraint on word processing. *Brain Research*, *1146*, 75–84. https://doi.org/10.1016/j.brainres.2006.06.101

Frank, S. L., Otten, L. J., Galli, G., & Vigliocco, G. (2015). The ERP response to the amount of information conveyed by words in sentences. *Brain and Language*, *140*, 1–11. https://doi.org/10.1016/j.bandl.2014.10.006

Kuperberg, G., Brothers, T., & Wlotko, E. W. (2020). A Tale of Two Positivities (and the N400): Distinct neural signatures are evoked by confirmed and violated predictions at different levels of representation. *Journal of Cognitive Neuroscience*, *32*(1), 12–35. https://doi.org/10.1162/jocn_a_01465

Kutas, M., & Hillyard, S. A. (1984). Brain potentials during reading reflect word expectancy and semantic association. *Nature*, *307*(5947), 161–163. https://doi.org/10.1038/307161a0

Stone, K., Nicenboim, B., Vasishth, S., & Rösler, F. (2020). Understanding the effects of constraint and predictability in ERP (Accepted stage 1 registered report). *Neurobiology of Language*.
